# Women’s Roles in Voluntary Medical Male Circumcision in Nyanza Province, Kenya

**DOI:** 10.1371/journal.pone.0044825

**Published:** 2012-09-19

**Authors:** Michele Lanham, Kelly L. L’Engle, Mores Loolpapit, Isaac Onyango Oguma

**Affiliations:** 1 Social and Behavioral Health Sciences, FHI 360, Research Triangle Park, North Carolina, United States of America; 2 Male Circumcision Consortium Project, FHI 360, Nairobi, Kenya; Indiana University and Moi University, United States of America

## Abstract

Women are an important audience for voluntary medical male circumcision (VMMC) communication messages so that they know that VMMC provides only partial protection against HIV. They may also be able to influence their male partners to get circumcised and practice other HIV protective measures after VMMC. This study was conducted in two phases of qualitative data collection. Phase 1 used in-depth interviews to explore women’s understanding of partial protection and their role in VMMC. Phase 2 built on the findings from the Phase 1, using focus groups to test VMMC communication messages currently used in Nyanza Province and to further explore women’s roles in VMMC. Sixty-four sexually active women between the ages of 18 and 35 participated. In Phase 1, all women said they had heard of partial protection, though some were not able to elaborate on what the concept means. When women in Phase 2 were exposed to messages about partial protection, however, participants understood the messages well and were able to identify the main points. In Phases 1 and 2, many participants said that they had discussed VMMC with their partner, and for several, it was a joint decision for the man to go for VMMC. These findings suggest that current VMMC messaging is reaching women, though communications could more effectively target women to increase their ability to communicate about partial HIV protection from VMMC. Also, women seem to be playing an important role in encouraging men to get circumcised, so reaching out to women could be a valuable intervention strategy for increasing VMMC uptake and promoting use of other HIV protective measures after VMMC.

## Introduction

Voluntary medical male circumcision (VMMC) provides partial HIV protection to men, reducing HIV acquisition by approximately 60 percent [1], [2], [3], [4]. Evidence is still emerging regarding the amount of HIV protection that VMMC provides to female partners. Early evidence did not show a direct protective effect of male circumcision on women’s HIV status [5]. However, observational studies show that female partners of circumcised men have lower HIV incidence than female partners of uncircumcised men [6]. Additionally, new mathematical models suggest that male circumcision may confer a 46 percent reduction in the rate of HIV transmission from men to their female partners, and that the benefits to women from VMMC may be larger and more important than originally anticipated [7].

Men must use other HIV protective measures with their female partners after VMMC to fully realize population-level benefits of VMMC on the HIV epidemic [5], [8], [9]. Communication messages about partial protection and the need to use other HIV protection methods are stressed in VMMC community education events and pre-procedure counseling as part of VMMC scale up in Nyanza Province, Kenya. Several organizations and agencies in Nyanza Province are providing information about VMMC through these channels as well as print media and radio. It is not clear, however, whether men and women understand messages about partial protection and the need to practice other HIV-protective behaviors after VMMC, since few studies have investigated this topic. One study conducted with men in Kenya found that participants understood that VMMC provided partial protection against HIV and other HIV protective measures were needed after VMMC, but few were able to accurately state the percentage reduction in HIV risk [10].

Although men are the main focus of VMMC education and information, female partners are also an important audience for two main reasons. First, understanding partial HIV protection from VMMC is essential so that women can make informed decisions about protecting themselves after a partner undergoes VMMC. While many women are hindered by gender inequalities and power differentials that limit their ability to negotiate safer sex [11], [12], some women may be able to sway their sexual partners to practice other HIV protective measures after VMMC. Secondly, female partners influence men’s decision to undergo medical circumcision [13], [14], [15] and focusing on them as a specific communication audience could help increase VMMC uptake, which has scaled up slower than anticipated in most priority countries [16].

The objectives of this study were to assess (1) women’s understanding of partial HIV protection from VMMC, (2) couples’ communication about VMMC, and (3) women’s engagement before and after the procedure. Women and men were recruited individually for study participation, but only data collected from women are presented here. The study was conducted in Nyanza Province, Kenya, a priority area for VMMC scale-up because of its high HIV prevalence (13.9 percent) and low male circumcision prevalence (45 percent), particularly among the Luo (22 percent), the predominant ethnic group in Nyanza [17].

## Methods

The study included two phases of qualitative data collection. In both phases, data collectors purposively recruited sexually active women between the ages of 18 and 35 in the urban district of Kisumu East and the more rural district of Siaya in Nyanza Province. Participants were recruited by the study’s data collectors through women-centered health clinics and by VMMC community mobilizers from the Nyanza Reproductive Health Society (NRHS). The community mobilizers recruited women through community outlets such as hair salons, through their male partners who were attending VMMC clinics for circumcision, and through other women who had already been interviewed for the study; mobilizers did not previously know the women they recruited. Data collectors were fluent in Dholuo (a local language) and English. They were trained in research ethics and qualitative and communication research methods. The study protocol was approved by the FHI 360 Protection of Human Subjects Committee in the United States and the Kenya Medical Research Institute (KEMRI) National Ethics Review Committee. Written informed consent was obtained from each study participant.

In Phase 1, 10 women with circumcised partners and 10 women with uncircumcised partners participated in individual in-depth interviews. This phase was designed to explore women’s understanding of partial protection and their role in VMMC, as there is little data on these topics. The semi-structured in-depth interview guide included questions about how participants would explain partial protection to others, the amount of HIV protection that VMMC provides to men and women, and the details of partner communication regarding circumcision. Questions about partial protection were general at the beginning, like “What have you heard about male circumcision and HIV?” and became more specific as the interview progressed, for example, “What does ‘partial protection’ mean to you?” and “What do you think it means that male circumcision reduces a man’s chances of getting HIV by 60%?”.

Phase 2 built on the findings from the Phase 1 and included testing communication messages and further exploring women’s roles in VMMC to inform current programs. Four focus groups were conducted with a total of 44 women (averaging 11 women per group). We recruited women whose male partners had undergone VMMC in the past six months, as these women are a priority audience for messages about partial protection, and they were most likely to provide detailed information about partner communication and women’s roles in VMMC. Focus groups assessed women’s understanding of partial protection by testing five printed VMMC communication materials that are currently used for demand generation and awareness raising in Nyanza Province. Three of the materials focused on partial protection, one highlighted the benefits of VMMC for women, and one discussed how women can support their partners before, during, and after VMMC. A character vignette about a fictional couple, Constance and Moses, was presented in the focus groups to explore partner communication before and after VMMC and women’s support of their partners throughout the VMMC process. Focus group participants were asked to draw on their own experiences and partnerships when answering the questions about Constance and Moses.

Data collectors audio recorded in-depth interviews and simultaneously translated and transcribed them verbatim into English [18]. For the focus group discussions, data collectors audio recorded them and wrote expanded notes immediately following the discussion, using notes taken during the discussions and the audio recordings [18]. Through a standard iterative process [19], a codebook was developed for each phase and used to structurally and thematically code the interviews and focus groups using QSR NVivo version 8. Two data analysts independently coded transcripts, and inter-coder agreement was assessed twice for each phase, using a percent agreement score [20]. Each time, the score was greater than 85 percent. The codebooks were updated after each inter-coder agreement check and as new themes emerged. Code reports were generated, and an inductive thematic analysis [21] was conducted to address the study objectives.

## Results

Sixty-four women participated in the study –20 women in Phase 1 and 44 in Phase 2. Participants were split almost equally between Kisumu East (n = 31) and Siaya (n = 33). Participants’ demographics were similar in Phases 1 and 2. The average age of participants was 25 in Phase 1 and 24 in Phase 2. The majority were married (15/20 in Phase 1, 34/44 in Phase 2) and did not work for money (12/20 in Phase 1, 30/44 in Phase 2). About half (11/20 in Phase 1, 23/44 in Phase 2) completed secondary school or higher.

### Understanding Partial Protection

All women in Phase 1 reported that they had heard that VMMC provides partial protection against HIV. Primary sources of information about VMMC were the radio and health facilities, including hospitals and HIV testing centers. Women said that the best way to reach them with messages about VMMC was through the radio, chief’s barazas (community meetings), and meetings at health centers. Women in Siaya specifically said that posters were not a good way to reach women because *“understanding was low*;” participants said this was particularly true among older women. They said that markets and women’s groups were good ways of specifically reaching women. Participants believed that healthcare workers and circumcised men were the most knowledgeable and trustworthy sources of information about VMMC because they had personal experience with it.

The most common way participants explained partial protection was using phrases like circumcision “reduces the risk of HIV,” provides “a little protection” or “some protection,” and “does not protect fully.” Other ways women described partial protection included the perceived mechanism of action (most often in terms of less dirt or germs and sometimes in terms of fewer cracks on the penis), percentages (such as VMMC “protects by 60 percent” or protection from VMMC is “not 100 percent”) and the need to use other protective measures after VMMC. When talking about post-VMMC HIV protective behaviors, only a few women mentioned specific behaviors like condom use and faithfulness to one sexual partner; others used more general phrases like “responsible behavior” and “any available protection.” A few women whose partners had been circumcised mentioned that they need to continue protecting themselves post-circumcision because they were not sure if their partners were faithful to them.

*“I will tell [my partner] that even after getting circumcised, he should still protect himself against HIV because circumcision does not offer full protection.”*

–Kisumu East participant, age 35, partner uncircumcised

*“If you get circumcised, the dirt that could have…the foreskin harbors a lot of dirt. After circumcision, the dirt that it harbors is gotten rid of, it becomes clean.”*

–Siaya participant, age 27, partner uncircumcised

*“What I heard about male circumcision when I attended a health talk. They said when our men go for circumcision, it protects HIV infection by 60 percent, and the remaining 40 percent, it’s still possible to get infected.”*

–Siaya participant, age 26, partner circumcised

Phase 1 participants were asked to specify by how much VMMC reduces a man’s chances of getting HIV. The most common response was a 60 percent reduction, and on a four-point scale (not at all, a little, a moderate amount, a lot), “a little” reduction was most frequently mentioned, followed by “a moderate amount” of reduction. Though all women said they had heard of partial protection, some were not able to elaborate about what the concept means. In addition, some women did not provide an explanation of the statement that VMMC reduces a man’s chances of getting HIV by 60 percent. These women did not have outright misunderstandings about the concepts of partial protection and 60 percent risk reduction but seemed unsure how to articulate their meaning. There were no discernible differences in descriptions of partial protection by partner’s circumcision status.

When asked whether a woman’s risk of HIV changes after her male partner gets circumcised, the majority of participants (16 out of 20) believed that female partners’ HIV risk would decrease. When asked why, several women described the perceived mechanism of action through which circumcision reduces the man’s HIV risk, reflecting participants’ belief that the HIV protection afforded to circumcised men is transferred directly to the female partner. Similarly, some simply stated that if a man’s risk was reduced, his female partner’s risk was also reduced. Other women talked about the man’s risk of HIV being reduced after VMMC and said that his wife’s chances of HIV also would be reduced because the man would be less likely to contract HIV in outside partnerships and then transmit it to his wife.

*“I’m thinking that if a man gets circumcised then it’s not easy for his sexual partner to get infected. If he doesn’t have the virus, the partner also does not have.”*

–Siaya participant, age 27, partner uncircumcised

Phase 2 built on the findings from the Phase 1 formative research by testing communications messages about partial protection currently used in VMMC programs in Nyanza. Phase 2 participants were able to accurately identify the main point of the three messages about partial protection: male circumcision does not provide full protection from HIV, and therefore other HIV protective measures must be used following VMMC. Participants appreciated that one of the materials mentioned that VMMC reduces the risk of HIV by 60 percent and that two materials mentioned specific ways of protecting against HIV after VMMC.

### Couples’ Communication and Women’s Engagement

The majority of women in Phase 1 whose partners were circumcised reported having discussed circumcision with their partners before he got circumcised. This included discussing the positive aspects of VMMC – in particular reduced HIV risk, reduced STI risk, and reduced penile problems like swelling. They also discussed the Luo tradition of not circumcising men and the experiences of people they knew who had been medically circumcised. Overall, women were supportive of VMMC and did not voice concern that her partner wanting to go for VMMC meant he wanted to continue or increase his risky sexual behaviors. Rather, women seemed pleased that this option for reducing HIV risk was available, and they liked the other benefits of VMMC including improved hygiene and fewer penile problems. A number of women said they encouraged their partners to get circumcised, and several women said it was a joint decision.

*“I told him that if he went for it was going to help him so it was good if he went for it. […] It was a joint decision.”*

–Siaya participant, age 21, partner uncircumcised

In discussions of the character vignette in Phase 2, participants in all focus groups said that Constance and Moses would discuss partial protection of VMMC, but there was disagreement regarding whether Constance and Moses would discuss the need to use other HIV protective measures following VMMC. Some said that the couple would discuss protective measures because they knew from VMMC counseling that circumcision only provides partial HIV protection. On the other hand, some participants said the couple would discuss HIV protective measures after VMMC only if there was a lack of trust in the relationship.

Among participants who said that Constance and Moses would discuss other protective measures, there was disagreement about what methods would be discussed. Participants in three groups said the couple would discuss and agree on faithfulness to one another. One group said that they would discuss the use of condoms, especially because condoms also prevent pregnancy and other sexually transmitted infections (STIs); however, other participants said condoms would not be considered because they can reduce erections in some men and have been known to burst. Only one group mentioned that partial protection and the need to use other HIV protective measures would be discussed *before* VMMC. Other groups indicated that this would be discussed after VMMC. The following are illustrative examples from expanded notes of focus group discussions.

*The participants then said after circumcision, Moses and Constance had to discuss the need to use other HIV prevention measures, and this is because male circumcision never offers 100 percent protection against HIV. They said the issue on faithfulness had to be agreed upon by both partners because even after male circumcision, one is still at 40 percent risk of contracting HIV. They also said condom use had to be mentioned because it does not only help prevention and spread of STIs, but also helps in preventing unwanted pregnancies.*

–Focus group, Siaya

*Participant 8 mentioned that she would only discuss other protective measures if there was lack of trust in the relationship. According to participant 6, she would not discuss other HIV protection measures if she personally knows that she is faithful to her partner, hence no need to use condom as a protective device.*

–Focus group, Kisumu East

When asked whether Constance would accompany Moses to VMMC services, focus group participants disagreed. Some said that Constance would go with Moses to demonstrate her support and commitment to him, and so that they would both benefit from VMMC counseling. Others said that VMMC is “a man’s thing” and that it would reflect badly on Moses if Constance accompanied him; in addition, some said that women are not allowed in VMMC surgical theaters. Ways that participants said Constance would support Moses after VMMC included not provoking Moses sexually during the abstinence period, helping him take care of his circumcision wound, providing healthy food, encouraging or accompanying Moses to the hospital for his wound check following VMMC, helping him with chores, and supporting him financially.

Phase 2 focus group participants responded very positively to two communications materials that talked about the benefits of VMMC for women and how women can support their partners before, during, and after VMMC. Participants appreciated the focus on women. After hearing these messages, participants stated they would talk with other women about the messages so other women would convince their uncircumcised partners to get circumcised.

## Discussion

### Partial Protection

Results from this study indicate that, for the most part, current VMMC messaging is reaching women and adequately communicating the meaning of partial protection, though women’s understanding could still be improved. There was little evidence that women have major misconceptions about the amount of protection that VMMC provides against HIV. Rather, many study participants were able to explain the meaning of partial protection and 60 percent risk reduction, though some had difficulty elaborating. For women who had difficulty elaborating on the meaning of partial protection, it seemed that this was due to lack of exposure to communication messages about partial protection rather than an inability to understand the concept of partial protection.

When women in Phase 2 were exposed to messages about partial protection in focus groups, they understood these messages well, were able to identify the main points and were conversational about partial protection and 60 percent risk reduction. Because some women in Phase 1 struggled to elaborate on the meaning of partial protection and 60 percent risk reduction, and women in Phase 2 were able to explain these concepts after being exposed to messages about them, our findings suggest that women’s exposure to messages could be improved. Reaching women through the channels they suggested – including markets, women’s groups, and health clinics – may be an effective way of increasing their exposure to VMMC messages.

The issue of protection afforded to women by VMMC could be more effectively addressed in VMMC communications. Though it is not yet known how much HIV protection VMMC provides to women, participants seemed to think they were afforded the same amount of protection as their male partners. Two other studies found that women had this same understanding [14]. This is a complicated concept to communicate accurately, but VMMC communications and counseling could emphasize that the amount of protection for women is not yet known, and the woman’s risk of HIV depends on her partner’s status and both partners’ sexual behavior.

### Couples’ Communication and Women’s Engagement

In both Phases 1 and 2, we found that most couples discussed VMMC, and women seem to be playing an important role in encouraging men to get circumcised. Reaching out to women could be a valuable intervention strategy for increasing VMMC uptake, especially among men ages 25–39, who have had lower rates of VMMC uptake in Nyanza Province [22], [23] but are more likely to be in steady relationships than younger men [17]. Also, given that partner communication has been found to play a key role in practicing HIV protective behaviors [24], [25], VMMC programs could also capitalize on the communication already taking place between partners regarding male circumcision to promote use of other HIV protective behaviors after VMMC, including promoting communication around condom use in steady relationships.

Phase 2 focus group participants indicated that other HIV protection would be discussed only if there was a lack of trust in the relationship, and few participants mentioned that they would discuss condom use. This is likely, in part, due to the association of condoms with infidelity [26] and the low use of condoms in steady relationships [27], [28]. To address this, communications messages could frame the use of other HIV protection after VMMC – including condoms – as part of a trusting relationship, in an attempt to normalize condom use among married couples. These messages could build on the positive step that couples have taken in seeking VMMC, for example, “Now that you’ve taken a first, important step to protect yourself AND your loved one(s) – it’s time to go that extra step. Go from partial to FULL protection! Always use condoms with the one you love.” Condoms could also be promoted for dual protection. One focus group mentioned this, and other studies have found that promoting condoms for dual protection against HIV and pregnancy might make condom use more acceptable in steady partnerships [29], [30], [31]. Finally, counseling, community education and mass media materials also could encourage couples to discuss *before* circumcision their plan for protecting themselves after VMMC.

Though women could benefit from HIV testing and VMMC counseling if they accompany their partners to VMMC services, many women seem to have the perception that VMMC services are only “a man’s thing.” Program implementers could endeavor to make VMMC services more couples centered and promote them as such because couples’ voluntary counseling and testing has been shown to increase spousal communication about HIV and increases the likelihood of disclosing one’s HIV status and practicing continued HIV protection after VMMC [24], [26]. VMMC service delivery presents a key opportunity to reach men and women in steady relationships with support to communicate about and practice HIV protective behaviors.

### Limitations and Conclusion

One limitation of this study is that one of the seminal male circumcision trials was conducted in Nyanza Province, so study participants may have higher awareness of VMMC and partial HIV protection, compared to women in other communities. In addition, study findings may not be as applicable in other settings where relationship dynamics differ, such as in South Africa where fewer women are married or living with a partner. Nevertheless, this study provides important insights into women’s knowledge of VMMC and partial protection, women’s VMMC communication needs, and opportunities to engage women in VMMC programs. The overall impact of VMMC programs on HIV incidence could be strengthened by more effectively involving women in VMMC uptake, HIV testing and VMMC counseling before the procedure, and promotion of abstinence and use of other HIV protective measures after VMMC. More research is needed on effective interventions for engaging women throughout the VMMC process, how to best promote condom use as part of a trusting relationship, and how to make VMMC services couples friendly.
